# Pro-adrenomedullin to predict severity and outcome in community-acquired pneumonia [ISRCTN04176397]

**DOI:** 10.1186/cc4955

**Published:** 2006-06-28

**Authors:** Mirjam Christ-Crain, Nils G Morgenthaler, Daiana Stolz, Christian Müller, Roland Bingisser, Stephan Harbarth, Michael Tamm, Joachim Struck, Andreas Bergmann, Beat Müller

**Affiliations:** 1 Department of Internal Medicine, University Hospital Basel, Switzerland; 2 Research Department, Brahms AG, Hennigsdorf, Germany; 3 Department of Pneumology, University Hospital Basel, Switzerland; 4 Division of Hospital Epidemiology, Geneva University Hospitals

## Abstract

**Introduction:**

Pro-adrenomedullin (proADM) is helpful for individual risk assessment and outcome prediction in sepsis. A major cause of sepsis is community-acquired pneumonia (CAP). The aim of this study was to investigate the value of proADM levels for severity assessment and outcome prediction in CAP.

**Methods:**

Data from 302 patients admitted to the emergency department with CAP were included in a prospective observational study. Procalcitonin, C-reactive protein levels, leukocyte count, clinical variables and the pneumonia severity index (PSI) were measured. ProADM levels were measured with a new sandwich immunoassay for mid regional ProADM (MR-proADM, Brahms AG, Hennigsdorf/Berlin, Germany).

**Results:**

ProADM levels, in contrast to C-reactive protein and leukocyte count, increased with increasing severity of CAP, classified according to the PSI score (ANOVA, *p *< 0.001). In patients who died during follow-up, proADM levels on admission were significantly higher compared to levels in survivors (2.1 (1.5 to 3.0) versus 1.0 (0.6 to 1.6) nmol/l, *p *< 0.001). In a receiver operating characteristic (ROC) analysis for survival, the area under the ROC curve (AUC) for proADM was 0.76 (95% confidence interval (CI) 0.71–0.81), which was significantly higher compared to procalcitonin (*p *= 0.004), C-reactive protein (*p *< 0.001) and total leukocyte count (*p *= 0.001) and similar to the AUC of the PSI (0.73, *p *= 0.54). A clinical model including the PSI and proADM increased the prognostic accuracy to predict failure compared to a model relying on the PSI alone (AUC, 0.77 (0.70 to 0.84), *p *= 0.03).

**Conclusion:**

ProADM, as a novel biomarker, is a useful tool for the risk stratification of patients with CAP.

## Introduction

Adrenomedullin (ADM) is one of the most potent vasodilating agents and has additional immune modulating, metabolic properties [1-4]. ADM also has a bactericidal activity that is further enhanced by modulation of complement activity and regulation [5-7]. Thus, it is not surprising that serum ADM levels are increased in sepsis [8]. The reliable measurement of ADM is challenging, since it is rapidly cleared from the circulation [1,2,9,10]. The more stable mid-region fragment of pro-adrenomedullin (proADM) directly reflects levels of the rapidly degraded active peptide ADM [11]. Recently, proADM has been shown to be a helpful prognostic tool for individual risk assessment in sepsis [12].

A main cause of sepsis is community-acquired pneumonia (CAP), which is the major infection-related cause of death in developed countries [13,14]. In the assessment and management of CAP, estimation of the disease severity is crucial for guiding therapeutic options such as the need for hospital or intensive care admission, the intensity of work-up, the choice and route of antimicrobial agents and the suitability for discharge [15,16].

The pneumonia severity index (PSI) is a widely accepted and validated severity scoring system that assesses the risk of mortality for pneumonia patients in a two-step algorithm [17]. However, its complexity is high, jeopardizing its dissemination and implementation, especially in everyday practice. Therefore, the CURB-65 score has been proposed as a simpler alternative [18]. Additionally, various easy to determine surrogate biomarkers have been proposed to predict disease severity in CAP patients, thereby aiming to complement the PSI score [19-21].

In this study, we investigated the prognostic value of proADM compared to other biomarkers (such as; procalcitonin, C-reactive protein (CRP) and leukocyte count), alone and in combination with the PSI in a well-defined cohort of 302 consecutive patients with CAP [22].

## Materials and methods

### Setting and study population

Data from 302 patients admitted to the emergency department with CAP were analyzed. The primary objective of the study was to evaluate antibiotic duration by procalcitonin guidance compared to standard recommended guidelines [22]. A predefined secondary endpoint was the assessment of prognostic factors and biomarkers in CAP.

Consecutive patients with CAP admitted from November 2003 through February 2005 to the University Hospital Basel, Switzerland, a 950 bed tertiary care hospital, were included. Patients had to be >18 years of age with a suspected CAP as principal diagnosis on admission. Excluded were patients with cystic fibrosis or active pulmonary tuberculosis, hospital-acquired pneumonia and severely immunocompromised patients. Patients were examined on admission to the emergency department by a resident supervised by a board-certified specialist in internal medicine. Baseline assessment included clinical data and vital signs, comorbid conditions, and routine blood tests. Functional status of the patients was assessed using a visual analogue scale, ranging from 0 (feeling extremely ill) to 100 (feeling completely healthy), and by a quality of life questionnaire for patients with respiratory illnesses [23].

CAP was defined by the presence of one or several of the following recently acquired respiratory signs or symptoms: cough, sputum production, dyspnea, core body temperature >38.0°C, auscultatory findings of abnormal breath sounds and rales, leukocyte count >10 or <4 × 10^9 ^cells l^-1 ^and an infiltrate on chest radiograph [14]. The PSI was calculated as described elsewhere [17]. Chest radiographs were screened by the physician in charge and reviewed by a senior radiologist, unaware of clinical and laboratory findings.

The study was approved by the local ethics committee for human studies and written informed consent was obtained from all patients.

### Outcome

All patients were followed-up for a mean duration of 6.9 ± 1.9 weeks [22]. At the follow-up visit, outcome was evaluated by clinical, laboratory, radiographic and microbiological criteria. Cure was defined as resolution of clinical, laboratory and radiographic signs of CAP. Improvement was defined as reduction of clinical signs and symptoms, improvement of laboratory findings (for example; CRP, procalcitonin and leukocyte count) and a reduction in the number or intensity of radiographic signs of CAP. Treatment success represented the sum of the rates for cure and improvement. Treatment failure included death, recurrence or persistence of clinical, laboratory and radiological signs of CAP at follow-up.

Patients who survived until follow-up were counted as survivors whereas patients who died within the follow-up period were counted as non-survivors.

### Microbial investigations

The laboratory workup for the patients with CAP has been previously described [22]. Briefly, it included sputum samples from Gram stain and culture, two blood samples for culture and a urine sample for detection of *Legionella pneumophila*.

### Measurement of proADM and other laboratory parameters

ProADM was detected in EDTA plasma of all patients with a new sandwich immunoassay (MR-proADM, BRAHMS AG, Hennigsdorf, Berlin, Germany), as described [24]. The assay (normal reference range 0.33 ± 0.7 nmol/l) has an analytical detection limit of 0.08 nmol/l and a functional assay sensitivity of 0.12 nmol/l. Procalcitonin was measured by a time-resolved amplified cryptate emission (TRACE) technology assay (Kryptor^® ^PCT, Brahms AG, Hennigsdorf, Berlin, Germany) with a functional assay sensitivity of 0.06 μg/l. CRP was measured with an enzyme immunoassay (EMIT, Merck Diagnostica, Zurich, Switzerland).

### Statistical analysis

Discrete variables are expressed as counts (percentage) and continuous variables as means ± standard deviation (SD) or median and interquartile range in parentheses unless stated otherwise. Frequency comparison was done by chi-square test. Two-group comparison of normally distributed data was performed by Students *t *test. For multigroup comparisons, one-way analysis of variance with least square difference for *post hoc *comparison was applied. For data not normally distributed, the Mann-Whitney *U *test was used if only two groups were compared and the Kruskal-Wallis one-way analysis of variance was used if more than two groups were being compared. Receiver-operating-characteristics were calculated using STATA (version 9, Statacorp, Texas, USA). Thereby, outcomes were either survival until follow-up, or failure including death until follow-up, respectively. To estimate the potential clinical relevance of proADM measurements, we used likelihood-ratio tests to determine whether logistic regression models that included measurements of proADM and the PSI provided a significant better fit than did logistic regression models limited to the PSI alone [25]. Correlation analyses were performed by using Spearman rank correlation. Levels that were non-detectable were assigned a value equal to the lower limit of detection for the assay. All testing was two-tailed and *p *values less than 0.05 were considered to indicate statistical significance.

## Results

### Patients

Detailed baseline characteristics of the study population are summarized in Table 1. The mean age of the 302 patients was 69.6 ± 17.0 years. Of the patients, 73 (24.2%) were smokers and 61 (20.2%) were pretreated with antibiotics. Fever >38°C was present in 60% of CAP patients and the typical triad of cough, fever and dyspnea, as reported by the patient, in 58% of cases. Overall, 87.5% of patients had relevant co-morbidities.

The mean PSI of all patients was 99.4 ± 35.3 points: 22 patients (7.3%) had a PSI class I; 41 (13.6%) a PSI class II; 57 (18.9%) a PSI class III, 130 (43.0%) a PSI class IV; and 52 (17.2%) a PSI class V. 271 patients (89.7%) were hospitalized for more than one night.

A microbiological diagnosis was achieved in 80 (26.5%) patients (in respiratory secretions in 51 (16.9%) and in blood cultures in 29 (9.6%) patients). The most frequently isolated microorganism was *Streptococcus pneumoniae *(detected in 42 patients, 14%), followed by *Pseudomonas aeruginosa *(10 patients, 3%), *Haemophilus influenzae *(7 patients, 2%), *Klebsiella pneumoniae *(5 patients, 2%), and *L. pneumophila *(5 patients, 2%).

### ProADM levels and severity of CAP

**Figure 1:**
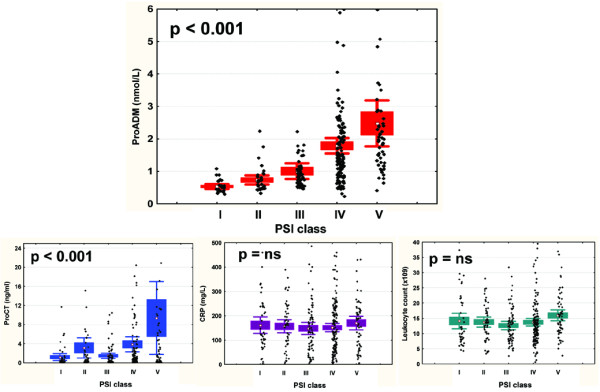
Pro-adrenomedullin (proADM), procalcitonin (proCT), C-reactive protein (CRP) levels and leukocyte count in different severities of community-acquired pneumonia. Data are shown as means ± standard error of the mean, with scatterplots representing all values. PSI, pneumonia severity index.

ProADM levels increased with increasing severity of CAP, classified according to the PSI score (*p *< 0.001). This gradual increase was also present but less pronounced for procalcitonin levels (*p *< 0.001), and not significant for CRP (*p *= 0.24), total leukocyte count (*p *= 0.13) (Figure 1), body temperature (*p *= 0.30) and the visual analogue scale (*p *= 0.39).

ProADM levels were significantly higher on admission (median (interquartile range) 1.1 (0.7 to 1.9) nmol/l compared to levels at follow-up after 6.9 ± 1.9 weeks (0.7 (0.5 to 1.0) nmol/l, p < 0.001). ProADM levels correlated with other biomarkers of infection, that is, procalcitonin (r = 0.51, *p *< 0.001), and to a lesser degree with CRP (r = 0.16, *p *< 0.01), and total leukocyte count (r = 0.23, *p *< 0.001). There was a significant correlation with the PSI score (r = 0.64, *p *< 0.001) and with serum creatinine levels (r = 0.60, *p *< 0.001).

ProADM levels were significantly higher in patients with multilobar pneumonia (1.4 (0.9 to 2.2) nmol/l) compared to patients with unilateral pneumonia (1.0 (0.6 to 1.8) nmol/l, *p *= 0.01). The respective values for procalcitonin were 0.8 (0.3 to 3.9) versus 0.5 (0.2 to 1.6), *p *= 0.02. CRP and leukocyte count were not significantly different between the two groups (data not shown). Patients with positive blood cultures had significantly higher proADM levels compared to patients with negative blood cultures (2.4 (1.6 to 3.0) versus 1.0 (0.6 to 1.7) nmol/l, *p *< 0.001). The respective values were: for procalcitonin, 8.0 (2.1 to 20.2) versus 0.4 (0.2 to 1.3), *p *< 0.001; for CRP, 197.5 (119.7 to 268.9) versus 122.7 (62.6 to 203.5), *p *= 0.002; and for leukocyte count, 17.1 ± 8.9 versus 13.2 ± 6.2, *p *= 0.004.

Patients who were hospitalized for more than one night had significantly higher proADM levels compared to patients who were not hospitalized or were hospitalized only for one night (1.1 (0.7 to 1.9) versus 0.73 (0.45 to 1.1) nmol/l, *p *= 0.001). The respective values were: for procalcitonin, 0.5 (0.2 to 2.6) versus 0.2 (0.1 to 0.77) μg/l, *p *= 0.002; for CRP (132.0 (65.5 to 211.8) versus 84.6 (40.0 to 190.0) mg/L, *p *= 0.052; and for leukocyte count, 13.4 ± 6.5 versus 14.5 ± 7.8 × 10^9^/l, *p *= 0.76.

### ProADM levels as a prognostic marker for outcome

At follow-up, 251 patients had a successful outcome (213 were cured, 38 improved). Failure at follow-up was noted in 51 patients (including death in 38 patients). Thus, the mortality rate was 12.6%.

In patients who died during follow-up, proADM levels on admission were significantly higher compared to levels in survivors (2.1 (1.5 to 3.0) versus 1.0 (0.6 to 1.6) nmol/l, *p *< 0.001). The respective values were: for procalcitonin, 0.7 (0.4 to 3.0) versus 0.4 (0.1 to 0.9) μg/l, *p *= 0.03); for CRP, 153 (93 to 204) versus 126.3 (63 to 211) mg/l, *p *= 0.57; and for total leukocyte count, 14.8 ± 8.2 versus 13.4 ± 6.4 × 10^9^/l, *p *= 0.24.

In a receiver operating characteristic (ROC) analysis where sensitivity was calculated with those patients who died until follow-up (*n *= 38) and specificity was assessed with those patients who survived until follow-up (*n *= 264), the area under the ROC curve (AUC) for proADM was 0.76, which was significantly better compared to procalcitonin (*p *= 0.004), CRP (*p *< 0.001) and total leukocyte count (*p *= 0.001) and similar to the AUC of the PSI (*p *= 0.54). The optimal prognostic accuracy for proADM was 1.8 nmol/l. With this cut-off, the sensitivity to correctly predict mortality until follow-up was 80%, the specificity 72%, the positive likelihood ratio (LHR+) 2.9 and the negative likelihood ratio (LHR-) 0.28. For the PSI with an optimal threshold of 101 points, the sensitivity was 58%, the specificity 84%, the LHR+ 3.7 and the LHR- 0.5.

**Figure 2:**
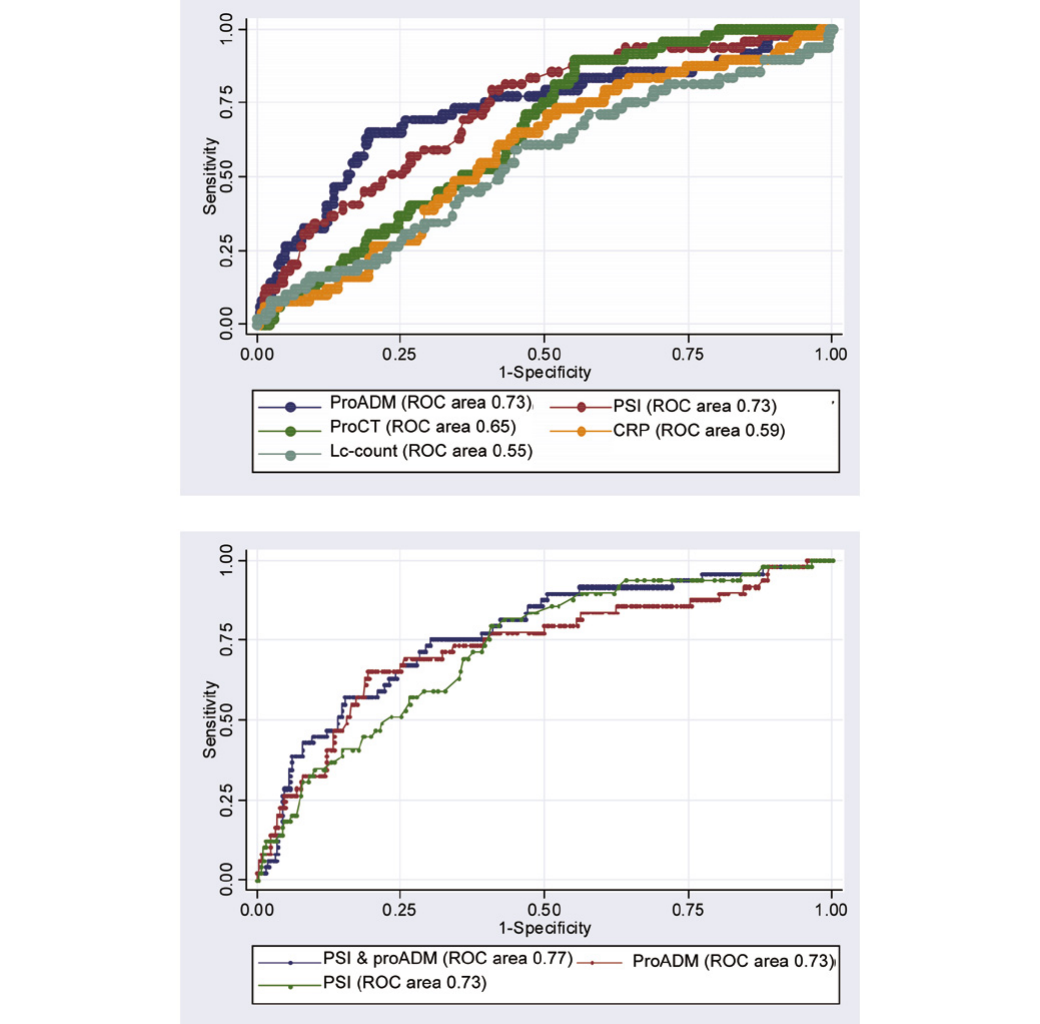
Receiver operator curve analysis of different laboratory parameters predicting failure after treatment of community-acquired pneumonia. Data on admission are shown. Upper panel: receiver operator curve (ROC) plot analysis of different parameters (i.e., pro-adrenomedullin (proADM), procalcitonin (proCT), C-reactive protein (CRP), leukocyte count (Lc count) and the pneumonia severity index (PSI)). Lower panel: ROC plot analysis of a combined model of proADM and the PSI compared to proADM and the PSI alone.

To predict failure including death, the AUC for proADM was 0.73 (0.68 to 0.78), which was significantly higher compared to CRP (AUC 0.59 (0.53 to 0.65), *p *= 0.02), and leukocyte count (0.55 (0.49 to 0.61), *p *= 0.002) and similar to the PSI (AUC 0.73 (0.67 to 0.78), *p *= 0.93) and procalcitonin (0.65 (0.59 to 0.70), *p *= 0.11) (Figure 2, upper panel).

Forty-one patients needed to be transferred to the ICU during hospitalization. To predict the need for ICU stay, proADM had an AUC of 0.65 (0.59 to 0.70), which was similar to the AUCs of CRP, leukocyte count, procalcitonin and the PSI (data not shown).

As a measure of clinical usefulness, we evaluated the combined role of proADM levels and the PSI as predictors of failure. ProADM could significantly improve the prognostic accuracy of the PSI to predict failure (AUC for the combined model, 0.77 (0.70 to 0.84), *p *= 0.03, compared to the PSI alone) (Figure 2, lower panel).

## Discussion

ProADM levels on admission predict the severity and outcome of CAP with a similar prognostic accuracy as the PSI and a higher prognostic accuracy compared to commonly measured clinical and laboratory parameters.

A key decision for a clinician is whether to admit a patient with CAP [26]. This decision is complex and depends on many variables, including estimates of the severity of illness. It often relies on the clinician's judgment; however, the interpretation of clinical signs and symptoms lacks standardization and validation and is prone to inter-observer variability [27]. In addition, physicians continue to be conservative and commonly overestimate the risk of death in patients with CAP [28]. Thus, prognostic scoring rules have been developed to predict severity of CAP and outcome, with the PSI being a well-validated prognostic classification score [17,18,29-31]. Limitations of the PSI include a potential overemphasis on age and the fact that for clinical ease, the PSI dichotomizes continuous values such as heart rate or oxygen saturation into normal and abnormal values. The intra-observer variation of the PSI is reported to be around 10%, with most patients misclassified in high-risk classes IV and V [32]. The PSI is better validated for assessing patients with a low mortality risk who may be suitable for home management rather than for those with severe CAP at the time of hospital admission [18]. Some clinicians argue that the score is not practical for routine patient management, restricting its widespread adoption. The CURB-65 score has been proposed as a simpler alternative; however, it had not been as extensively validated [18]. The American Thoracic Society (ATS) guidelines do not offer any algorithm for the clinical assessment of disease severity [14,33]. There are also no universally accepted criteria for severe CAP requiring admission to an ICU.

In this context, there is need for readily measurable biomarkers predicting the severity level and outcome of CAP. ProADM levels on admission had a similar prognostic accuracy as the PSI and, based on our data, represent an additional and easy-to-determine prognostic tool. It is advisable to support the complex task of prognostic assessment and treatment decisions with several clinical and laboratory parameters that may mirror different physiological aspects. ProADM might also act as an additional margin of safety to guide management decisions, since adding proADM to the PSI increased predictive accuracy.

CRP was put forward as a useful marker for predicting disease severity in patients with pneumonia [19]. In contrast, in our study, CRP could not differentiate between different severities of CAP, as defined by the PSI. It must be taken into account that CRP is a rather non-specific marker of acute-phase inflammation and, therefore, is subject to the influence of many other factors. IL-6, a key stimulator of hepatic CRP release, has also been investigated for the determination of the severity of CAP [34]. Measuring of plasma cytokines like IL-6, however, is cumbersome, partly because of the short plasma half-life and the presence of blocking factors [35]. Most recently, D-Dimers have been suggested as a prognostic parameter in CAP [21]. As a limitation of our study, we did not measure D-Dimer levels and can not show comparative results. Procalcitonin has been proposed as a marker of disease severity by our group and others [20,22]. However, based on our results, proADM is a prognostic marker and predicts the severity of disease, whereas procalcitonin is rather a diagnostic tool able to guide decisions on antibiotic therapy [22,36].

Two main mechanisms might be responsible for the increase of circulating proADM in infections, including CAP. Firstly, as a member of the calcitonin gene family, ADM is widely expressed and extensively synthesized during severe infections, that is, sepsis, similar to other calcitonin peptides, namely procalcitonin and calcitonin-gene related peptides [37]. Our data demonstrate that proADM levels are also increased in milder forms of infection like pneumonia, which can be regarded as a precursor of sepsis. Bacterial endotoxins and proinflammatory cytokines up-regulate ADM gene expression in many tissues, both *in vitro *and *in vivo *in rodents and humans [38,39]. In addition, a decreased clearance by the kidneys may be responsible in part for the increased proADM levels in infections [8]. This hypothesis is also supported by a significant correlation between proADM and creatinine levels in patients enrolled in our study. An alternative site of clearance of proADM may be the lung. It has been reported that ADM concentrations from the aorta are slightly lower than those from the pulmonary artery during selective catheter sampling [40]. Therefore, impaired removal of circulating ADM during pulmonary circulation resulting from infection-associated lung injury may partly contribute to the elevation of plasma ADM levels [12].

Circulating levels of the potent mediator ADM are kept within a very narrow range in order to prevent harmful excessive effects. Hence, even in sepsis, circulating levels of ADM are only modestly elevated, and are not significantly different between patients with systemic inflammatory response syndrome and patients with sepsis, prohibiting its use as a diagnostic and prognostic tool. In contrast, circulating levels of less active precursor peptides are less tightly controlled and, therefore, have a much higher diagnostic and prognostic range. Our finding of an ADM precursor facilitates the assessment of the actual release of ADM gene products under pathological conditions and thereby improves the diagnostic and prognostic accuracy.

Some limitations of our study merit consideration. First, proADM measurements were done as a predefined secondary endpoint [22]. Future intervention studies should be encouraged to evaluate proADM levels as a prognostic tool in CAP and other infections. Second, since the etiology remained unidentified in a considerable proportion of cases because of the low sensitivity of conventional microbiological tests, we cannot make any conclusion about the usefulness of proADM to predict the etiology of CAP.

A single biomarker will always oversimplify the interpretation of important variables and, therefore, proADM is meant to complement, rather than to supersede, clinician's judgment and/or validated severity scores. Besides clinical judgment, social factors and patient preferences will also influence where and how to manage CAP.

## Conclusion

ProADM is a novel biomarker that seems to be a useful tool for the risk stratification of patients with CAP. Accurate and objective models of prognosis for CAP will help physicians to assess a patient's risk profile and improve the decisions about hospitalization and treatment.

## Key messages

• 	In patients with CAP, mid-regional proADM levels on admission can predict outcome, with a similar prognostic accuracy as the PSI score.

• 	ProADM, used in conjunction with the PSI, can improve the prognostic accuracy to predict failure compared to a model relying on the PSI alone.

## Abbreviations

ADM = adrenomedullin; AUC = area under the curve; CAP = community-acquired pneumonia; CRP = C-reactive protein; LHR = likelihood ratio; PSI = pneumonia severity index; ROC = receiver operating characteristic.

## Competing interests

BM has served as consultant and received payments from Brahms (the manufacturer of pro-adrenomedullin assay) to attend meetings related to the trial and for travel expenses, speaking engagements, and research. SH has received speaker honoraria from Brahms. NM, JS and AB are employees of Brahms. All other co-authors declare that they have no competing interests.

## Authors' contributions

BM had the idea for the study and directed study design, data collection and analysis and writing of the report. MCC drafted the protocol, collected and analyzed data, and wrote the report. NM did the analyses and helped in analyzing and writing of the report. DS, RB, CM, SH and MT had substantial contributions in planning of the study, data collection, interpretation of data and/or writing of the manuscript. JS and AB had a substantial role in the analyses.

## Figures and Tables

**Table 1:** Baseline characteristics of the 302 patients

Characteristic	
Age, years	69.6 ± 17.0^a^
Male sex, no. (%)	187 (61.9)
Smoking status	
Current smoker, no. (%)	73 (24.2)
Pack-years for smokers	40.1 ± 24.2^a^
Antibiotic pretreatment (%)	61 (20.2)
Coexisting illnesses, no. (%)	
Coronary artery disease	97 (32.1)
Hypertensive heart disease	78 (25.8)
Congestive heart failure	16 (5.3)
Peripheral vascular disease	20 (6.6)
Cerebrovascular disease	16 (5.3)
Renal dysfunction	81 (26.8)
Liver disease	31 (10.3)
Diabetes mellitus	61 (20.2)
Chronic obstructive pulmonary disease	76 (25.2)
Neoplastic disease	48 (15.9)
History, no. (%)	
Cough	270 (89.4)
Sputum	221 (73.2)
Dyspnea	229 (75.8)
Examination, no. (%)	
Rales	271 (89.7)
Laboratory findings	
CRP (mg/l), median (IQ range)	129.7 [65–211]
Procalcitonin (μg/l), median (IQ range)	0.5 [0.2–2.2]
Leukocyte count (× 10^9^)	13.6 ± 6.7^a^
proADM (pmol/l), median (IQ range)	1.1 [0.6–1.9]
Radiographic findings, no. (%)	
Pleural effusion	37 (12.3)
Multilobar CAP	53 (17.5)
PSI points	99.4 ± 35.3^a^
PSI class, no. (%)	
I, II and III	120 (39.7)
IV	130 (43)
V	52 (17)

^a^Values are means ± standard deviation unless stated otherwise. Because of rounding, percentages may not sum to 100. ADM, adrenomedullin; CRP, C-reactive protein; PSI = pneumonia severity index.

## References

[B1] Hinson JP, Kapas S, Smith DM (2000). Adrenomedullin, a multifunctional regulatory peptide. Endocr Rev.

[B2] Eto T (2001). A review of the biological properties and clinical implications of adrenomedullin and proadrenomedullin N-terminal 20 peptide (PAMP), hypotensive and vasodilating peptides. Peptides.

[B3] Kitamura K, Sakata J, Kangawa K, Kojima M, Matsuo H, Eto T (1993). Cloning and characterization of cDNA encoding a precursor for human adrenomedullin. Biochem Biophys Res Commun.

[B4] Linscheid P, Seboek D, Zulewski H, Keller U, Muller B (2005). Autocrine/paracrine role of inflammation-mediated calcitonin gene-related peptide and adrenomedullin expression in human adipose tissue. Endocrinology.

[B5] Pio R, Martinez A, Unsworth EJ, Kowalak JA, Bengoechea JA, Zipfel PF, Elsasser TH, Cuttitta F (2001). Complement factor H is a serum-binding protein for adrenomedullin, and the resulting complex modulates the bioactivities of both partners. J Biol Chem.

[B6] Marutsuka K, Nawa Y, Asada Y, Hara S, Kitamura K, Eto T, Sumiyoshi A (2001). Adrenomedullin and proadrenomudullin N-terminal 20 peptide (PAMP) are present in human colonic epithelia and exert an antimicrobial effect. Exp Physiol.

[B7] Martinez A, Pio R, Zipfel PF, Cuttitta F (2003). Mapping of the adrenomedullin-binding domains in human complement factor H. Hypertens Res.

[B8] Hirata Y, Mitaka C, Sato K, Nagura T, Tsunoda Y, Amaha K, Marumo F (1996). Increased circulating adrenomedullin, a novel vasodilatory peptide, in sepsis. J Clin Endocrinol Metab.

[B9] Jougasaki M, Burnett JC (2000). Adrenomedullin: potential in physiology and pathophysiology. Life Sci.

[B10] Kato J, Tsuruda T, Kitamura K, Eto T (2003). Adrenomedullin: a possible autocrine or paracrine hormone in the cardiac ventricles. Hypertens Res.

[B11] Struck J, Tao C, Morgenthaler NG, Bergmann A (2004). Identification of an Adrenomedullin precursor fragment in plasma of sepsis patients. Peptides.

[B12] Christ-Crain M, Morgenthaler NG, Struck J, Harbarth S, Bergmann A, Muller B (2005). Mid-regional pro-adrenomedullin as a prognostic marker in sepsis: an observational study. Crit Care.

[B13] Mortensen EM, Coley CM, Singer DE, Marrie TJ, Obrosky DS, Kapoor WN, Fine MJ (2002). Causes of death for patients with community-acquired pneumonia: results from the Pneumonia Patient Outcomes Research Team cohort study. Arch Intern Med.

[B14] Niederman MS, Mandell LA, Anzueto A, Bass JB, Broughton WA, Campbell GD, Dean N, File T, Fine MJ, Gross PA (2001). Guidelines for the management of adults with community-acquired pneumonia. Diagnosis, assessment of severity, antimicrobial therapy, and prevention. Am J Respir Crit Care Med.

[B15] Bartlett JG, Dowell SF, Mandell LA, File TM, Musher DM, Fine MJ (2000). Practice guidelines for the management of community-acquired pneumonia in adults. Infectious Diseases Society of America. Clin Infect Dis.

[B16] Mandell LA, Marrie TJ, Grossman RF, Chow AW, Hyland RH (2000). Canadian guidelines for the initial management of community-acquired pneumonia: an evidence-based update by the Canadian Infectious Diseases Society and the Canadian Thoracic Society. The Canadian Community-Acquired Pneumonia Working Group. Clin Infect Dis.

[B17] Fine MJ, Auble TE, Yealy DM, Hanusa BH, Weissfeld LA, Singer DE, Coley CM, Marrie TJ, Kapoor WN (1997). A prediction rule to identify low-risk patients with community-acquired pneumonia. N Engl J Med.

[B18] Lim WS, van der Eerden MM, Laing R, Boersma WG, Karalus N, Town GI, Lewis SA, Macfarlane JT (2003). Defining community acquired pneumonia severity on presentation to hospital: an international derivation and validation study. Thorax.

[B19] Almirall J, Bolibar I, Toran P, Pera G, Boquet X, Balanzo X, Sauca G (2004). Contribution of C-reactive protein to the diagnosis and assessment of severity of community-acquired pneumonia. Chest.

[B20] Masia M, Gutierrez F, Shum C, Padilla S, Navarro JC, Flores E, Hernandez I (2005). Usefulness of procalcitonin levels in community-acquired pneumonia according to the patients outcome research team pneumonia severity index. Chest.

[B21] Querol-Ribelles JM, Tenias JM, Grau E, Querol-Borras JM, Climent JL, Gomez E, Martinez I (2004). Plasma d-dimer levels correlate with outcomes in patients with community-acquired pneumonia. Chest.

[B22] Christ-Crain M, Stolz D, Bingisser R, Muller C, Miedinger D, Huber PR, Zimmerli W, Harbarth S, Tamm M, Muller B (2006). Procalcitonin-guidance of antibiotic therapy in community-acquired pneumonia – a randomized trial. Am J Respir Crit Care Med.

[B23] Christ-Crain M, Jaccard-Stolz D, Bingisser R, Gencay MM, Huber PR, Tamm M, Muller B (2004). Effect of procalcitonin-guided treatment on antibiotic use and outcome in lower respiratory tract infections: cluster-randomised, single-blinded intervention trial. Lancet.

[B24] Morgenthaler NG, Struck J, Alonso C, Bergmann A (2005). Measurement of midregional proadrenomedullin in plasma with an immunoluminometric assay. Clin Chem.

[B25] Harbarth S, Holeckova K, Froidevaux C, Pittet D, Ricou B, Grau GE, Vadas L, Pugin J (2001). Diagnostic value of procalcitonin, interleukin-6, and interleukin-8 in critically ill patients admitted with suspected sepsis. Am J Respir Crit Care Med.

[B26] Aronsky D, Dean NC (2001). How should we make the admission decision in community-acquired pneumonia?. Med Clin North Am.

[B27] Wipf JE, Lipsky BA, Hirschmann JV, Boyko EJ, Takasugi J, Peugeot RL, Davis CL (1999). Diagnosing pneumonia by physical examination: relevant or relic?. Arch Intern Med.

[B28] McIvor RA (2004). Plasma d-dimer for outcome assessment in patients with CAP: not a replacement for PSI. Chest.

[B29] Farr BM, Sloman AJ, Fisch MJ (1991). Predicting death in patients hospitalized for community-acquired pneumonia. Ann Intern Med.

[B30] Fine MJ, Singer DE, Hanusa BH, Lave JR, Kapoor WN (1993). Validation of a pneumonia prognostic index using the MedisGroups Comparative Hospital Database. Am J Med.

[B31] Garcia-Ordonez MA, Garcia-Jimenez JM, Paez F, Alvarez F, Poyato B, Franquelo M, Colmenero JD, Juarez C (2001). Clinical aspects and prognostic factors in elderly patients hospitalised for community-acquired pneumonia. Eur J Clin Microbiol Infect Dis.

[B32] Aronsky D, Haug PJ (2000). Assessing the quality of clinical data in a computer-based record for calculating the pneumonia severity index. J Am Med Inform Assoc.

[B33] Niederman MS, Craven DE (2005). Guidelines for the management of adults with hospital-acquired, ventilator-associated, and healthcare-associated pneumonia. Am J Respir Crit Care Med.

[B34] Kolsuz M, Erginel S, Alatas O, Alatas F, Metintas M, Ucgun I, Harmanci E, Colak O (2003). Acute phase reactants and cytokine levels in unilateral community-acquired pneumonia. Respiration.

[B35] Luna CM (2004). C-reactive protein in pneumonia: let me try again. Chest.

[B36] Christ-Crain M, Muller B (2005). Procalcitonin in bacterial infections – hype, hope, more or less?. Swiss Med Wkly.

[B37] Becker KL, Nylen ES, White JC, Muller B, Snider RH (2004). Clinical review 167: Procalcitonin and the calcitonin gene family of peptides in inflammation, infection, and sepsis: a journey from calcitonin back to its precursors. J Clin Endocrinol Metab.

[B38] Shoji H, Minamino N, Kangawa K, Matsuo H (1995). Endotoxin markedly elevates plasma concentration and gene transcription of adrenomedullin in rat. Biochem Biophys Res Commun.

[B39] Linscheid P, Seboek D, Zulewski H, Keller U, Muller B (2005). Autocrine/paracrine role of inflammation-mediated Calcitonin Gene-Related Peptide and Adrenomedullin expression in human adipose tissue. Endocrinology.

[B40] Nishikimi T, Kitamura K, Saito Y, Shimada K, Ishimitsu T, Takamiya M, Kangawa K, Matsuo H, Eto T, Omae T (1994). Clinical studies on the sites of production and clearance of circulating adrenomedullin in human subjects. Hypertension.

